# Thermal Tolerance Data and Molecular Identification Are Useful for the Diagnosis, Control and Modeling of Diseases Caused by *Thielaviopsis paradoxa*

**DOI:** 10.3390/pathogens12050727

**Published:** 2023-05-17

**Authors:** Abiodun Abeeb Azeez, Daniel Ofeoritse Esiegbuya, Emad Jaber, Wenzi Ren, Adebola Azeez Lateef, Amarachi Ojieabu, Fred O. Asiegbu

**Affiliations:** 1 Department of Forest Sciences, University of Helsinki, Latokartanonkaari 7, P.O. Box 27, 00014 Helsinki, Finland; 2 Rainforest Research Station, Forestry Research Institute of Nigeria (FRIN), Jericho Hill, Ibadan P.M.B 5054, Nigeria; 3 Pathology Division, Nigerian Institute for Oil Palm Research (NIFOR), Benin City P.M.B 1030, Nigeria; 4 Crop Protection Department, PNG Oil Palm Research Association (PNGOPRA), Dami Research Station, Kimbe P.O. Box 97, Papua New Guinea; 5 Department of Plant Biology, Faculty of Life Sciences, University of Ilorin, Ilorin P.M.B 1515, Nigeria

**Keywords:** *Ceratocystis paradoxa*, disease management, growth rate, optimum temperature, pathogen

## Abstract

Several economically important diseases of forest trees and agricultural crops in many parts of the world have been linked to the ascomycete fungal pathogen *Thielaviopsis paradoxa*. This study compared the growth rate of 41 isolates of *T. paradoxa* sourced from different hosts and two countries (Nigeria and Papua New Guinea (PNG)) under six temperature levels (22 °C, 25 °C, 30 °C, 32 °C, 34 °C and 35 °C). Phylogenetic relationships were obtained from the analysis of their nuclear ribosomal DNA internal transcribed sequence (ITS) data. While all the isolates from PNG and few from Nigeria grew optimally between 22 °C and 32 °C, the majority had their highest growth rate (2.9 cm/day) between 25 °C and 32 °C. Growth performances were generally low between 34 °C and 35 °C; no isolate from the sugar cane grew at these high temperatures. The oil palm isolate DA029 was the most resilient, with the highest growth rate (0.97 cm/day) at 35 °C. Phylogenetic analysis delineated five clusters: a very large clade which accommodates the majority (30 Nigerian and 3 PNG oil palm isolates) and four small clades containing two members each. To a large extent, the clustering pattern failed to address the temperature–isolate relationship observed. However, only the four small clades represent isolates with similar temperature tolerances. It is most likely that wider and robust analyses with more diverse isolates and genetic markers will provide better insight on thermal resilience of *T. paradoxa*. Additionally, future research to establish relationships between vegetative growth at different temperatures and of different pathogenicity and disease epidemiology merits being explored. The results might provide useful information for the formulation of effective management and control strategies against the pathogen, especially in this era of climate change.

## 1. Introduction

Among the key players (host, pathogen, biotic and environmental factors) commonly highlighted in the disease triangle model, environmental factors play the most significant role in the development of diseases [1,2]. The germination of spores into infective structures for colonizing substrates precedes the development of a disease under suitable environmental conditions such as temperature [3]. Temperature is a critical environmental factor required for effective germination of conidia and growth of mycelia, influences the development and severity of diseases and has been regarded as an essential parameter to be monitored for effective biocontrol of plant diseases [4,5]. Although fungi are unable to control their internal temperature, they survive under climatic conditions with a diverse range of temperatures and exhibit varying growth rate and metabolism in the presence of other necessary environmental conditions such as moisture and water. The knowledge of growth behavior of fungi with respect to temperature changes is paramount and an important aspect of fungal physiology [6]. Many research reports have described high temperatures greater than 25 °C as the optimum temperature for stimulation of enzymatic activity and growth of germ tubes as well as infective apparatus for several filamentous fungal species [7,8]. Impacts of environmental factors on microbial activities such as growth are often studied by in vitro experimentation or in situ assessment of the samples under natural conditions [9]. There is an increasing evidence that temperature plays significant role on growth behavior and pathogenicity of plant pathogens [10,11]. These observations can be explored for the control of plant pathogens. In fungal-like-organisms such as *Phytophthora* spp., thermal intensity influences mycelial production, spore formation and disease development [12,13,14]. Shelley et al. [15] demonstrated the significance of temperature on the formation of sporangia and zoospore cyst from the early stage of disease cycle to the late stage of infection caused by *Phytophthora kernoviae* in *Rhododendron ponticumin* and *Annoma cherimolya*. Trecate et al. [16] reported in vitro inhibition of germination mechanisms and pathogenicity of the two causal agents of cucurbit powdery mildew, *Podosphaera xanthii* and *Golovinomyces orontii*, at 35 °C and recommended the finding for prediction of the disease severity under field conditions. Increased temperature and host resistance have been reported to reduce the fitness of the causal agent of the crown disease of wheat, *Fusarium pseudograminearum* [17], with a similar necrotrophic lifestyle as *Thielaviopsis paradoxa*.

The cosmopolitan and diverse family of plant pathogens Ceratocystidaceae consists of many pathogenic fungi of economic importance. These include the six well studied members of the genus *Thielaviopsis* (*T. cerberus*, *T. ethacetica*, *T. euricoi*, *T. musarum, T. paradoxa* and *T. punctulata*) formerly recognized as *Ceratocystis paradoxa* complex [18,19] causing diseases in a wide range of hosts. These fungal species are characterized by specialized hyphae with tips bearing two types of conidia, the small philiadic endoconidia and large pigmented aleurioconidia [20]. *Thielaviopsis paradoxa* de Seynes is a filamentous ascomycete, wound pathogen and soil-borne fungus that attacks all parts of its host plants [21,22] which include pineapple (*Ananas comosus*), coconut (*Cocos nucifera*) [23], sugarcane (*Saccharum officinarum*) [24], cocoa (*Theobroma cacao*), oil palm (*Elaeis guineensis*) and date palm (*Araecu catheru*) [25]. It is widely distributed in nature and has been identified as the causative agent of several economically important diseases of monocotyledonous plants such as leaf spot, fruit rot and butt rot of pineapple, leaf spot, bud rot, heart rot and root decay of coconut, pineapple disease of sugar cane, black scorch disease of date palm and dry basal rot of oil palm [23,25,26]. *Thielaviopsis paradoxa* has long been associated with bending of apical region (also known as neck bending disease) in date palms in the Arabian Peninsula as well as many other countries where date palms are cultivated [27,28]. Recently, it has also been linked to neck bending disease of oil palm since its first discovery in 2019 in the southern part of Nigeria where it caused 65–70% mortality in young oil palms (2–3 years) on plantations [29,30].

The previous phylogenetic studies of some species in the genus *Thielaviopsis* categorized the taxon according to geographical origin into four distinct clades: the North American [31], Latin American [32,33], African [19,34] and Asian-Australian clades [31,35,36]. Similarly, there is growing evidence from DNA sequence studies that *Ceratocystis*, the recognized sexual states of *Thielaviopsis* (according to the dual nomenclature system), consist of species complexes with marked phylogenetic lineages with members sharing related eco-morphological characteristics making them worthy of being considered distinct genera [31,37,38]. Identification of fungi based on morphological and physiological characteristics was the foremost approach in fungal systematics until the advent of PCR-based molecular techniques. PCR has solved the inherent problems with the traditional classification including the unique nature of fungi which poses a tremendous problem to the phenotype-based classification [39]. In recent times, several molecular techniques for fungal identification have been reported. These include fluorescent in situ hybridization (FISH), denaturing gradient gel electrophoresis (DGGE), DNA array hybridization, pulse-field gel electrophoresis (PFGE), terminal restriction fragment length polymorphism (T-RFLP) and DNA sequencing-based techniques which are the most frequently used [40,41,42]. There is continued interest in the use of DNA barcoding as an identification tool for fungi species due to its fastness and accuracy [43,44]. The use of a rDNA-internal transcribed spacer (ITS) as a DNA barcode for intraspecific fungi has received considerable attention in the recent time due to the conservativeness of its sequence and ability to explore abundant site variabilities [45,46]. Many research works have documented the use of ITS and other molecular markers in resolving taxonomy issues and for phylogenetic reconstruction in many species of organisms including fungi. Alvarez et al. [47] studied the genetic diversity of some strains of *Thielaviopsis paradoxa* from Ecuador, Colombia and Brazil using random amplified polymorphic DNA (RAPD) markers and PCR sequencing of the internal transcribed spacer (ITS) region of 5.8 S ribosomal DNA (rDNA) and found that the population is predominantly clonal. Similarly, Borges et al. [48] were able to characterize *T. ethacetica* isolates based on phylogenetic analyses of both ITS and TEF-1 amplified gene sequences whereas the analysis of the sexual and asexual phases of the isolates with morphological markers failed to confirm the identity of the fungus. Parsimony analyses of the sequences of the ITS and the large subunit (LSU) of the nuclear rDNA have recognized some *Thielaviopsis* species as a monophyletic group. Among these species, only *T. populi*, *T. ovoidea* and *T. thielavioides* which show identical morphology have been revealed to be discreet by the analyses of their rDNA sequences [20].

The increasing awareness of *T. paradoxa* as a causal agent of many diseases of monocots with varying level of severity across their growing regions globally necessitates characterization and critical study of this pathogen with respect to its physiological and molecular diversity. Therefore, this study was designed to (i) study the growth behavior of *T. paradoxa* isolates from oil palm, date palm and sugar cane in Nigeria and Papua New Guinea with respect to some selected temperature levels, (ii) determine the optimum temperature for the growth of *T. paradoxa*, (iii) identify isolates of *T. paradoxa* that are resilient to high temperature and (iv) establish a phylogenetic relationship among the isolates using sequence data of PCR- amplified ITS genes.

## 2. Materials and Methods

### 2.1. Fungal Isolates

**Figure 1:**
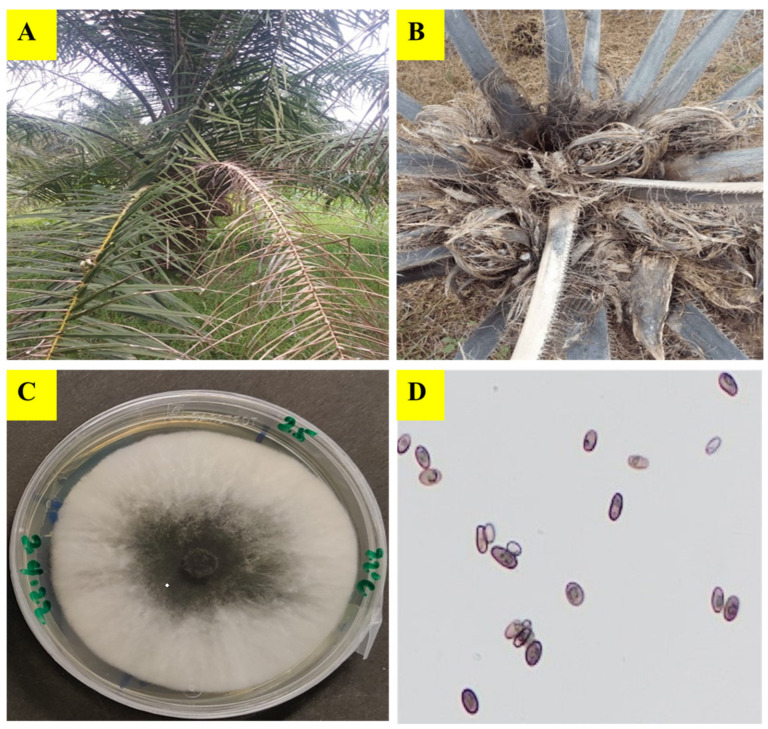
A dry basal rot-infected oil palm showing fracture of the lower fronds (**A**). A cross section of a dry basal rot-infected oil palm showing fruit bunch rotting symptom (**B**). A pure culture of *Thielaviopsis paradoxa* (**C**). Microscopic view of two ascospore types produced by *T. paradoxa* (**D**).

Forty-one isolates of *T. paradoxa* used for this experiment were collected from Nigeria and Papua New Guinea (PNG) (Table 1). Koch’s postulate has been previously established for these isolates. The Nigerian samples were isolated from oil palm trunks showing symptoms of dry basal rot (Figure 1A,B) and/neck bending disease (see Figures 1–4 in [29]) and soil samples, date palm fruits and sugar cane stems within three states (Edo, Delta and Jigawa) in Nigeria. The samples collected from Edo state consisted of twenty-one oil palm trunk samples from four sampling points (6°46′05.9″ N 6°29′45.5″ E, 6°46′0.01″ N 5°51′39.5″ E, 6°46′08.7″ N 5°51′04.3″ E and 6°46′14.5″ N 5°50′45.1″ E) within an oil palm plantation at Uhiere, seven soil samples from Ugbowo (6°39′69.3″ N 5°60′92.02″ E) and two soil samples from Udo (5°28′39.3″ N 8°05′07.2″ E). Isolates were also obtained from five sugar cane stems and one rotten date fruit bought from a market in Asaba (Delta State) and Dutse (Jigawa State) respectively. The isolation was carried out at the laboratory of Nigerian Institute for Oil palm Research located in Benin City, Edo State, Nigeria. Isolated samples were preserved on Potato Dextrose Agar (PDA) slant in 2.0 mL Eppendorf tubes at room temperature.

Five fungal samples collected in PNG were from the Papua New Guinea Oil Palm Research Association (PNGOPRA) herbarium. The samples were isolated from degraded felled oil palm trunks from two sites (Waigani estate and Hagita estate) within the New Britain Palm Oil Ltd. (NBPOL) plantations at Milne Bay Province. The wood degradation trials for felled oil palm trunks was carried out as a PNGOPRA, CABI Bioscience and Birkbeck College collaborative project during 1998–2001. A pure culture of each isolate (Figure 1C) was prepared and verified under microscope (Figure 1D) at the Department of Forest Sciences laboratory, Viikki, Helsinki, before physiological and molecular studies. Voucher specimen of the 41 isolates of *T. paradoxa* were deposited at the Hambi Culture Collection, (https://www.helsinki.fi/en/infrastructures/biodiversity-collections/infrastructures/microbial-domain-biological-resource-centre-hambi (accessed on 16 January 2023), Department of Microbiology, University of Helsinki.

### 2.2. Radial Growth Studies

The effect of temperature on the growth of the isolates of *T. paradoxa* was investigated under six temperature levels (22 °C, 25 °C, 30 °C, 32 °C, 34 °C and 35 °C) maintained in the incubator (Model: Binder KB53). Each isolate was grown in a 9 cm Petri dish containing potato dextrose agar (PDA) and incubated at 25 °C for 3 days. For each temperature, a mycelial plug (5 mm in diameter) was cut from area around the edge of the plate and used to inoculate the center of a freshly prepared Petri dish containing PDA and kept in a designated temperature in the incubator. Each isolate was prepared in two replicates. Daily observation of the surface radial growth for each isolate was carried out by measuring the distance reached by mycelium on two perpendicular diameters pre-drawn at the base of each plate [49,50]. This was conducted for three consecutive days after inoculation.

### 2.3. DNA Extraction, Amplification and Sequencing

Fresh cultures of the isolates were pre-grown on cellophane membrane overlaid on PDA and incubated at 25 °C for 3 days. Genomic DNA was extracted from the fungal hyphae according to the modified CTAB procedure reported by Terhonen et al. [51]. Fungal hyphae were harvested, homogenized in a micro pestle and transferred to a 2 mL Eppendorf tube already placed in liquid nitrogen. After homogenization, 900 µL of CTAB extraction buffer (pre-heated at 65 °C) and 9 µL of DTT were added to the sample. The tube was vortexed and incubated at 65 °C for 15 min. An equal volume of Chloroform: Isoamyl alcohol (IAA) (24:1) was added. The tube was shaken vigorously and centrifuged at 10,000 rpm for 10 min. The supernatant (700 µL) was transferred to another 2 mL Eppendorf tube; an equal volume of Chloroform: Isoamyl alcohol (24:1) was added and centrifuged at 10,000 rpm for 10 min. The supernatant (500 µL) was transferred to a new 1.5 mL tube. DNA was precipitated by adding one volume of ice-cold isopropanol, leaving the tube on ice for 15 min and centrifuging at 12,000 rpm at 4 °C for 10 min. The supernatant was discarded, and DNA pellet was washed by adding 100 µL of 70% cold ethanol at room temperature and centrifuging at 12,000 rpm at 4 °C for 5 min. The supernatant was discarded, and the pellet was re-suspended in 50 µL of nuclease-free water. The DNA quality was measured using nanodrop.

PCR-amplification of the ITS region and sequencing were carried out at StarSEQ Gmbh, Germany. The PCR reactions were conducted in an XP BIOER Technology thermal cycler (Hangzhou China). Phusion polymerase was used according to the recommended procedure by ThermoFisher Scientific, Helsinki, Finland. ITS1 F and ITS4 were used as primers. The PCR mix (20 µL) contained 1 µL of genomic DNA (10 ng/µL), 0.2 µL of Phusion polymerase (2 U), 1 µL of each primer (10 µM), 0.4 µL of dNTPs (10 mM), 4 µL of buffer (5 X) and 12.4 µL of nuclease free water. Thermal cycling condition for the amplification was 98 °C for 30 s for initial denaturation, 35 cycles for 10 s each for denaturation at 98 °C, annealing at 55 °C for 10 s, extension at 72 °C for 30 s and final extension at 72 °C for 10 min [52]. The PCR products obtained were run on 1% agarose and amplified bands, compared with Thermofisher Scientific DNA ladder 1 kb and purified with GenElute^TM^ PCR clean-up kit (Sigma-Aldrich, St. Louis, MO, USA). The ITS amplicons were sequenced with the ITS4 primer used for amplifying the DNA by PCR [53].

### 2.4. Statistical Analysis

The mean values obtained for the growth diameter for the isolates at the selected temperatures were used to calculate absolute growth rates (cm/day) of the isolates. The data were first subjected to normality and homogeneity tests [5], and one-way analysis of variance (ANOVA) was used to ascertain whether there were statistically significant differences. Mean comparison was done with Tukey-test (*p* < 0.01). All the tests were conducted in R studio version 4.1.3.

### 2.5. Phylogenetic Analysis

The initial analysis of ITS rDNA sequences of the 41 isolates of *T. paradoxa* was conducted on the BLAST interface of the NCBI website (https://www.ncbi.nlm.nih.gov/) (accessed on 24 December 2022). Additionally, two ITS rDNA sequences of two related fungal species, *Ceratocystis fimbriata* and *Thielaviopsis thielavioides*, retrieved from gene bank were used as an outgroup for comparative analysis. The gene bank sequence with the highest identity for each of the query sequence was obtained. Multiple sequence alignment and manual editing of the sequences were performed with Aliview version 1.28. Phylogeny was inferred based on Bayesian analysis using the Markov chain Monte Carlo (MCMC) method performed in MrBayes version 3.2.7 [54]. The best-fitting likelihood model TVM+G among the four evolutionary models revealed by JModelTest [55] was selected for the analysis. There was an incremental heating scheme that ensured four MCMC chains were run simultaneously for more than 150 K generations with a burn-in time of 50 K generations and a starting random tree [56]. The MrBayes output file created was used to generate the consensus tree with credibility values (percentage posterior probabilities (PP)) using the iTOL online tool (https://itol.embl.de/) (accessed on 12 April 2023).

## 3. Results

### 3.1. Temperature versus Isolate Growth Rate

The growth rates (cm/day) obtained three days after inoculation for each studied temperature is presented in Table 2. The maximum growth rate (2.93 cm d^−1^) was found in all the isolates except two sugar cane isolates, AA034 (2.07 cm d^−1^) and AA036 (2.23 cm d^−1^). Optimum temperatures for growth for the majority of the isolates lay between 25 °C and 32 °C.

At 22 °C, the growth rate ranging from 0.53 cm d^−1^ (AA036) to 2.93 cm d^−1^ was found in all the five PNG oil palm isolates and only eight from Nigeria. Between 25 °C and 30 °C, most of the isolates attained their maximum growth rate; exceptions were found in AA033 (2.75 cm d^−1^), AA034 (2.07 cm d^−1^) and AO36 (1.83 cm d^−1^) at 25 °C, AA032 (2.47 cm d^−1^), AA034 (1.47 cm d^−1^) and AA036 (2.23 cm d^−1^) at 30 °C and DA023 (2.6 cm d^−1^), AO32 (1.37 cm d^−1^), AO34 (1.40 cm d^−1^) and AO36 (1.20 cm d^−1^) at 32 °C. Conversely, at 34 °C, most of the isolates grew at comparatively lower rate which varied from 0.2 cm d^−1^ (AA032) and 2.03 cm d^−1^ (DA019). All the isolates had their least growth rate, and no visible growth was observed in the sugar cane isolates at 35 °C. However, the highest growth rate (0.97 cm d^−1^) recorded at this temperature was found in DA029. At all the temperatures, there were statistically significant differences between the growth rates of isolates, although the differences were much higher at 30 °C (F(40,41) = 275,700, *p* < 0.001) and 32 °C (F(40,41) = 214,215, *p* < 0.001).

Overall, the optimum temperature for all the isolates from PNG is between 22 °C and 32 °C while that of Nigerian isolates is between 25 °C and 32 °C. Isolates from oil palm were found to be more tolerant to higher temperatures compared to those from date fruit and sugar cane.

### 3.2. Phylogenetic Analysis of ITS Sequences

**Figure 2:**
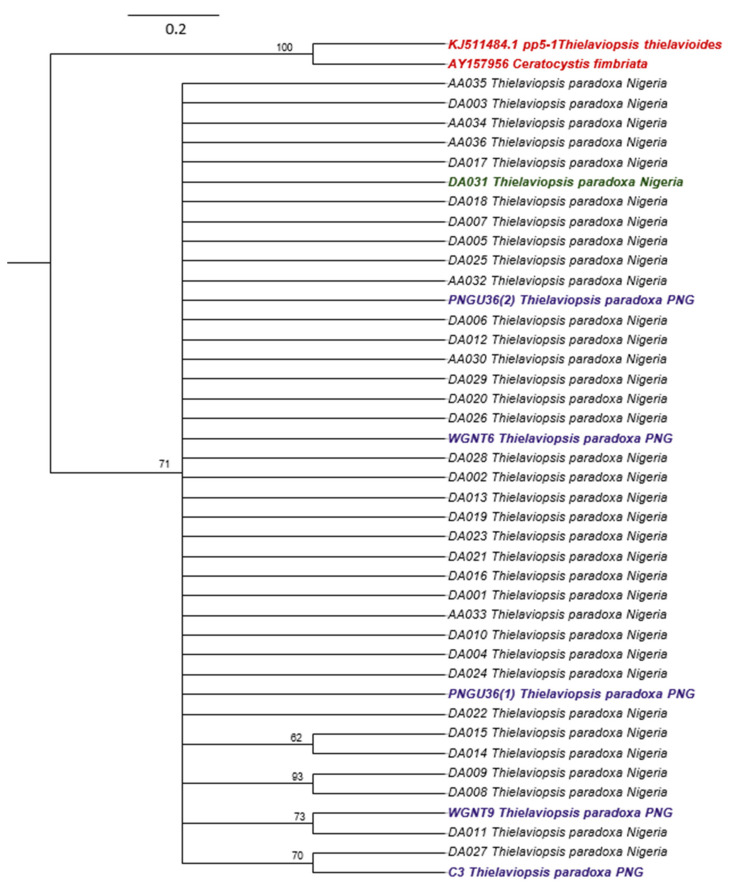
Phylogenetic tree constructed by Bayesian analysis of ITS rDNA sequences of 41 *Thielaviopsis paradoxa* isolates and two related species, *Ceratocystis fimbriata* and *T. thielavioides* (serving as outgroup; written in red colour). Numbers indicated above the branches are the relevant %PP values for the consensus tree. The scale bar represents the number of nucleotide substitutions per site. Isolates are written with black (oil palm isolates from Nigeria), purple (oil palm isolates from PNG), green (date fruit isolate from Nigeria) and blue (sugarcane isolates from Nigeria) colours.

A total of 41 ITS rDNA sequences were generated from the PCR-amplification of the genomic DNA of the isolates with ITS4 primers. The length of the sequences varied between 516 and 528 bp, and the percentage identity shared with *T. paradoxa*, according to the gene bank, varied between 99.02 and 100%. The 41 ITS sequences of *T. paradoxa* isolates and the two of *Ceratocystis fimbriata* and *Thielaviopsis thielavioides* were used to generate a Bayesian tree. The credibility values (percentage posterior probabilities (PP)) of the branching pattern of the phylogenetic tree are greater than 60% which indicates more than average level of reliability of the expressed relationship among the isolates (Figure 2). There was no definite clustering pattern indicative of origin, hosts, and substrates in the formation of the clades. The isolates formed five clusters: a large clade consisting of 30 Nigerian (oil palm, date fruit and sugar cane) and three PNG oil palm isolates and four small clades consisting of two members each. The clustering pattern evinced by the phylogram failed to address the temperature–isolate relationship observed in this study. While the large clade accommodated different categories of isolates with varied growth responses to temperatures, the four small clades represent members with similar temperature tolerances.

## 4. Discussion

The behavior of certain fungal species under laboratory conditions tends to correlate with their geographical distribution, seasonal occurrence and evolutional history [57]. This study was designed to evaluate the growth behavior of *T. paradoxa* isolates under different temperature regimes, characterize available isolate collections using rDNA ITS marker and establish a relationship between the temperature, growth and molecular data of the isolates.

Many studies from different parts of the world have reported various temperatures as optimum for growth of *T. paradoxa*. However, discrepancies exist in these reports. Our study has clearly demonstrated that a certain level of variability exists among the *T. paradoxa* isolates with respect to optimum temperature requirement for growth. These differential growth abilities found in some of the tested temperatures varied with source of isolates and geographical distribution. All the temperatures encouraged mycelial growth of the fungal isolates from Nigeria and PNG isolated from oil palm and date palm. While all isolates from PNG and a few from Nigeria grew optimally between 22 °C and 32 °C, the majority had their best growth performance between 25 °C and 32 °C. Meanwhile, some reports documented temperature ranges of 28–32 °C [58,59], 25–31 °C [57] and 25–30 °C [60] as optimal for growth of the fungus. Temperatures between 28 and 32 °C have been linked with high level of pathogenicity of the fungus in sugarcane [47]. We found the growth to be at the lowest rate between 34 °C and 35 °C and no visible mycelial proliferation and spore germination in the sugar cane isolates at these temperatures. Our finding, therefore, differs to some extent from a recent study which reported that *T. paradoxa* requires a temperature range of 15–35 °C for optimal vegetative growth [61]. Although this present study is only limited to the temperature range tested, it could be imperfect to compare other similar studies that reported temperature 12 °C and 36 °C as minimum and maximum [57] and ranges of 21–22 °C and <10 °C as optimal and inert, respectively, [62] for active mycelial growth and spore germination. Oil palm and date palm isolates performed relatively better compared to sugar cane isolates, with no observable growth in the latter at 35 °C. The oil palm isolate DA029 is obviously the most resilient at this temperature. Such occurrence of higher temperature tolerance in oil palm isolates could be due to a host–pathogen co-evolutionary relationship as these hosts are usually found in tropical regions characterized with high temperature. The physiological information provided in this study might be essential for efficient prediction of the possible effects of climate change on the epidemiology of diseases caused by *T. paradoxa* [63]. Additionally, the significant variabilities in thermal resistance exhibited by the isolates can influence the choice of strain selection in the formulation of biological control measures, although the relationship between the in vitro temperature–vegetative growth relationship and disease epidemiology must be first investigated [49].

As the findings in this study are based on laboratory observations, the results may not correspond perfectly to what may be observed under natural conditions [64], and further experiments in the presence of the host may be advisable. Nevertheless, our observation that *T. paradoxa* isolates from oil palm and date palm exhibited some tolerance to higher temperature compared to the sugarcane isolates could provide explanations to an unresolved mechanism that the pathogen attacks certain host species in the presence of others under suitable environmental conditions especially in tropical countries where temperature may reach a favorable level for the fungus to exhibit its pathogenicity. So far, most of the reported cases of the dry basal rot and neck bending disease in West African countries including Nigeria occurred when the atmospheric temperatures were close to the optimum temperature for vegetative growth of the fungus observed in this study. Therefore, regulating the temperature of the environment to an unfavorable level for the survival of the pathogen during the host susceptibility period may be applicable in the prevention of certain diseases such as the neck bending disease of oil palm caused by *T. paradoxa*. This could be through provision of temporary shading trees such as *Gliricidia sepium* used for shade management in *Theobroma cacao* [65]. This system will not only check excessive sunlight radiation which may increase the atmospheric temperature of the plant site but also contribute to soil nitrogen fixation. Soil nitrogen has recently been linked with *T. paradoxa* disease suppression [30]. The reported cases of *T. paradoxa* diseases within and across the oil palm growing countries so far vary with respect to severity. This phenomenon could be attributed to variation in temperature in these regions as supported by our observation that all the PNG isolates attained the maximum growth rate at 22 °C compared to only few isolates from Nigeria. Thus, considering such environments where the prevailing temperatures are unfavorable for *Thielaviopsis paradoxa* disease occurrence for cultivation of any susceptible host species may also be a good management strategy to curtail the disease spread. Even though, other factors such as host population density, genetic constitution and tolerance may also have a significant impact on pathogenicity [15]. This study will be a source of significant data on *T. paradoxa* disease forecast models for predicting the population dynamics of the pathogen as well as its disease incidence and severity.

The polymorphic nature and massive reference sequences of ITS markers in gene sequence banks have been variously explored in resolving taxonomic issues and gene population studies especially in fungi [42,66]. In this study, information provided by the ITS sequence BLAST on the NCBI interface showed a high-percentage identity of the isolates being shared with the existing *T. paradoxa* data in the gene bank which further confirms the presence of the pathogen in all the diseased tissues and soil samples where isolations were carried out. This molecular identification thus provides easier and more reliable alternative for identifying the pathogen [67] compared to an in vivo test which is more difficult to accomplish because successful inoculation of the pathogen requires wounding of tissue and aseptic isolation of the pathogen to avoid contamination. The phylogenetic analysis of the ITS sequence data confirmed that all the isolates were *T. paradoxa*, and limited variation was observed. The genetic closeness is obvious irrespective of origin, host, tissue or substrate from where the fungi were isolated, and the isolates could therefore be inferred to have emerged from a single population which reproduces primarily by vegetative means. Apart from the mode of reproduction, the cultural methods adopted for disease control, which has also been implicated with the spread, may be responsible for the lack of geographical structuring in the phylograms. A recent genetic diversity study carried out in South America using ITS and Random Amplified Polymorphic DNA (RAPD) markers has also suggested *T. paradoxa* isolates to be a clonal population [47]. However, the present study shows there may be at least five ITS alleles in worldwide populations of this pathogen. This ITS variability should be taken into consideration when designing molecular assays for the diagnosis of the pathogen.

A wider and more robust sampling capturing more sources of the pathogen as well as investigation with more genetic markers could improve understanding of the temperature–isolate growth relationship and the ITS region of *T. paradoxa*. This is especially important to determining the isolates of the pathogen that are resistant to higher temperature and can withstand climatic change. Additionally, research work to establish the relationship between in vitro growth response of the isolates to temperature changes and pathogenicity as well as disease epidemiology may be warranted to provide insights on development of effective management and control strategies for disease eradication.

## Figures and Tables

**Table 1:** Sources of isolates used.

S/N	Isolate ID	Host	Substrate	Origin	GPS Coordinate	Name of Collectors	Hambi Reference Number	Genebank Accession Number
1	DA001	Oil palm	Trunk	Uhiere, Nigeria	6°46′05.9″ N, 6°29′45.5″ E	D. O Esiegbuya and A. Ojieabu	2737	OQ422120
2	DA002	Oil palm	Trunk	Uhiere, Nigeria	6°46′05.9″ N, 6°29′45.5″ E	D. O Esiegbuya and A. Ojieabu	2736	OQ422121
3	DA003	Oil palm	Trunk	Uhiere, Nigeria	6°46′05.9″ N, 6°29′45.5″ E	D. O Esiegbuya and A. Ojieabu	2738	OQ422122
4	DA004	Oil palm	Trunk	Uhiere, Nigeria	6°46′05.9″ N, 6°29′45.5″ E	D. O Esiegbuya and A. Ojieabu	2739	OQ422123
5	DA005	Oil palm	Trunk	Uhiere, Nigeria	6°46′05.9″ N, 6°29′45.5″ E	D. O Esiegbuya and A. Ojieabu	2740	OQ422124
6	DA006	Oil palm	Trunk	Uhiere, Nigeria	6°46′05.9″ N, 6°29′45.5″ E	D. O Esiegbuya and A. Ojieabu	2741	OQ422125
7	DA007	Oil palm	Trunk	Uhiere, Nigeria	6°46′0.01″ N, 5°51′39.5″ E	D. O Esiegbuya and A. Ojieabu	2742	OQ422126
8	DA008	Oil palm	Trunk	Uhiere, Nigeria	6°46′0.01″ N, 5°51′39.5″ E	D. O Esiegbuya and A. Ojieabu	2743	OQ422127
9	DA009	Oil palm	Trunk	Uhiere, Nigeria	6°46′0.01″ N, 5°51′39.5″ E	D. O Esiegbuya and A. Ojieabu	2744	OQ422128
10	DA010	Oil palm	Trunk	Uhiere, Nigeria	6°46′0.01″ N, 5°51′39.5″ E	D. O Esiegbuya and A. Ojieabu	2745	OQ422129
11	DA011	Oil palm	Trunk	Uhiere, Nigeria	6°46′0.01″ N, 5°51′39.5″ E	D. O Esiegbuya and A. Ojieabu	2746	OQ422130
12	DA012	Oil palm	Trunk	Uhiere, Nigeria	6°46′0.01″ N, 5°51′39.5″ E	D. O Esiegbuya and A. Ojieabu	2747	OQ422131
13	DA013	Oil palm	Trunk	Uhiere, Nigeria	6°46′08.7″ N, 5°51′04.3″ E	D. O Esiegbuya and A. Ojieabu	2748	OQ422132
14	DA014	Oil palm	Trunk	Uhiere, Nigeria	6°46′08.7″ N, 5°51′04.3″ E	D. O Esiegbuya and A. Ojieabu	2749	OQ422133
15	DA015	Oil palm	Trunk	Uhiere, Nigeria	6°46′08.7″ N, 5°51′04.3″ E	D. O Esiegbuya and A. Ojieabu	2750	OQ422134
16	DA016	Oil palm	Trunk	Uhiere, Nigeria	6°46′08.7″ N, 5°51′04.3″ E	D. O Esiegbuya and A. Ojieabu	2751	OQ422135
17	DA017	Oil palm	Trunk	Uhiere, Nigeria	6°46′14.5″ N, 5°50′45.1″ E	D. O Esiegbuya and A. Ojieabu	2752	OQ422136
18	DA018	Oil palm	Trunk	Uhiere, Nigeria	6°46′14.5″ N, 5°50′45.1″ E	D. O Esiegbuya and A. Ojieabu	2753	OQ422137
19	DA019	Oil palm	Trunk	Uhiere, Nigeria	6°46′14.5″ N, 5°50′45.1″ E	D. O Esiegbuya and A. Ojieabu	2754	OQ422138
20	DA020	Oil palm	Trunk	Uhiere, Nigeria	6°46′14.5″ N, 5°50′45.1″ E	D. O Esiegbuya and A. Ojieabu	2755	OQ422139
21	DA021	Oil palm	Trunk	Uhiere, Nigeria	6°46′14.5″ N, 5°50′45.1″ E	D. O Esiegbuya and A. Ojieabu	2756	OQ422140
22	DA022	Oil palm	Soil	Ugbowo, Nigeria	6°39′69.3″ N, 5°60′92.02″ E	D. O Esiegbuya and A. Ojieabu	2757	OQ422141
23	DA023	Oil palm	Soil	Ugbowo, Nigeria	6°39′69.3″ N, 5°60′92.02″ E	D. O Esiegbuya and A. Ojieabu	2758	OQ422142
24	DA024	Oil palm	Soil	Ugbowo, Nigeria	6°39′69.3″ N, 5°60′92.02″ E	D. O Esiegbuya and A. Ojieabu	2759	OQ422143
25	DA025	Oil palm	Soil	Ugbowo, Nigeria	6°39′69.3″ N, 5°60′92.02″ E	D. O Esiegbuya and A. Ojieabu	2760	OQ422144
26	DA026	Oil palm	Soil	Ugbowo, Nigeria	6°39′69.3″ N, 5°60′92.02″ E	D. O Esiegbuya and A. Ojieabu	2761	OQ422145
27	DA027	Oil palm	Soil	Ugbowo, Nigeria	6°39′69.3″ N, 5°60′92.02″ E	D. O Esiegbuya and A. Ojieabu	2762	OQ422146
28	DA028	Oil palm	Soil	Ugbowo, Nigeria	6°39′69.3″ N, 5°60′92.02″ E	D. O Esiegbuya and A. Ojieabu	2763	OQ422147
29	DA029	Oil palm	Soil	Udo, Nigeria	5°28′39.3″ N, 8°05′07.2″ E	D. O Esiegbuya and A. Ojieabu	2764	OQ422148
30	AA030	Oil palm	Soil	Udo, Nigeria	5°28′39.3″ N, 8°05′07.2″ E	A. A Azeez	2765	OQ422149
31	DA031	Date palm	Fruit	Dutse, Nigeria	11°18′79.3″ N, 9°55′17.2″ E	D. O Esiegbuya and A. Ojieabu.	2735	OQ422150
32	AA032	Sugar cane	Stem	Asaba, Nigeria	5°29′21.32″ N, 6°00′14..42″ E	A. A Azeez	2766	OQ422151
33	AA033	Sugar cane	Stem	Asaba, Nigeria	5°29′18.32″ N, 6°00′04.62″ E	A. A Azeez	2767	OQ422152
34	AA034	Sugar cane	Stem	Asaba, Nigeria	5°29′54.37″ N, 6°00′17.64″ E	A. A Azeez	2734	OQ422153
35	AA035	Sugar cane	Stem	Asaba, Nigeria	5°29′33.72″ N, 6°00′28.01″ E	A. A Azeez	2735	OQ422154
36	AA036	Sugar cane	Stem	Asaba, Nigeria	5°29′26.11″ N, 6°00′33.34″ E	A. A Azeez	2768	OQ422155
37	C3	Oil palm	Trunk	MBE, Papua New Guinea	Unknown	B. Ritchie and PNGOPRA team	2769	OQ422156
38	WGNT6	Oil palm	Trunk	MBE, Papua New Guinea	Unknown	B. Ritchie and PNGOPRA team	2771	OQ422160
39	WGNT9	Oil palm	Trunk	MBE, Papua New Guinea	Unknown	B. Ritchie and PNGOPRA team	2772	OQ422159
40	PNGU36(1)	Oil palm	Trunk	MBE, Papua New Guinea	Unknown	B. Ritchie and PNGOPRA team	2773	OQ422157
41	PNGU36(2)	Oil palm	Trunk	MBE, Papua New Guinea	Unknown	B. Ritchie and PNGOPRA team	2774	OQ422158

**Table 2:** Growth rates (cm/day) of *Thielaviopsis paradoxa* isolates at different temperature three days after inoculation.

Isolate	22 °C	25 °C	30 °C	32 °C	34 °C	35 °C
DA001	2.93 a	2.93 a	2.93 a	2.75 abc	1.08 BC	0.17 OP
DA002	2.17 jklmn	2.93 a	2.93 a	2.93 a	1.57 tuvwxy	0.30 KLMNO
DA003	2.93 a	2.93 a	2.93 a	2.93 a	1.70 rstuvw	0.40 GHIJKLMNO
DA004	2.47 bcdef	2.93 a	2.93 a	2.93 a	1.73 qrstu	0.67 GHIJKLMN
DA005	2.75 ab	2.93 a	2.93 a	2.93 a	1.63 rstuvwxy	0.33 JKLMNO
DA006	2.53 cdefgh	2.93 a	2.93 a	2.93 a	1.73 rstuv	0.67 EFG
DA007	2.93 a	2.93 a	2.93 a	2.93 a	1.73 rstuv	0.70 DEFGH
DA008	2.80 abc	2.93 a	2.93 a	2.93 a	1.83 pqrst	0.50 FGHIJK
DA009	2.93 a	2.93 a	2.93 a	2.93 a	1.77 qrstu	0.37 HIJKLMN
DA010	2.32 ghijkl	2.93 a	2.93 a	2.93 a	1.07 BC	0.23 MNOP
DA011	2.93 a	2.93 a	2.93 a	2.93 a	1.80 pqrst	0.50 FGHIJKLM
DA012	2.77 abc	2.93 a	2.93 a	2.93 a	1.90 nopqr	0.67 EFG
DA013	2.93 a	2.93 a	2.93 a	2.93 a	1.5 uvwxyz	0
DA014	2.53 cdefgh	2.93 a	2.93 a	2.93 a	1.67 rstuvwx	0.67 EFGH
DA015	2.8 abc	2.93 a	2.93 a	2.93 a	1.67 rstuvwx	0.37 IJKLMNO
DA016	2.93 a	2.93 a	2.93 a	2.93 a	1.70 rstuvw	0.27 LMNOP
DA017	2.36 fghijk	2.93 a	2.93 a	2.93 a	1.70 rstuvw	0.30 KLMNO
DA018	2.20 ijklm	2.93 a	2.93 a	2.93 a	1.67 rstuvwx	0.40 GHIJKLMNO
DA019	2.16 jklmn	2.93 a	2.93 a	2.93 a	2.03mnopq	0.27 LMNOP
DA020	2.93 a	2.93 a	2.93 a	2.93 a	1.87 opqrs	0.63 EFGHI
DA021	2.40 efghij	2.93 a	2.93 a	2.93 a	1.70 rstuvw	0.67 EFG
DA022	2.30 ghijklm	2.93 a	2.93 a	2.93 a	1.77 qrstu	0.37 IJKLMNO
DA023	2.16 jklmn	2.93 a	2.93 a	2.60 bcdef	1.23 zAB	0
DA024	1.80 pqrst	2.93 a	2.93 a	2.93 a	1.43 wxyzA	0.43 GHIJKLMNO
DA025	2.53 cdefgh	2.93 a	2.93 a	2.93 a	1.63 rstuvwxy	0.63 EFGHI
DA026	2.67 abcde	2.93 a	2.93 a	2.93 a	1.70 rstuvw	0.47 GHIJKLMN
DA027	2.53 cdefg	2.93 a	2.93 a	2.93 a	1.77 qrstu	0.43 GHIJKLMNO
DA028	2.40 efghij	2.93 a	2.93 a	2.93 a	1.70 rstuvw	0.60 EFGHIJ
DA029	2.70 abcd	2.93 a	2.93 a	2.93 a	1.60 stuvwxy	0.97 BCD
AA030	2.12 klmno	2.93 a	2.93 a	2.93 a	1.47 vwxyzA	0.23 MNOP
DA031	2.60 bcdef	2.93 a	2.93 a	2.93 a	1.37 yzA	0.47 GHIJKLMN
AA032	1.85 opqrs	2.93 a	2.46 defghi	1.36 yzA	0.20 NOP	0
AA033	1.63 rstuvwxy	2.75 abc	2.93 a	2.93 a	1.67 rstuvwx	0
AA034	1.47 vwxyzA	2.07 lmnop	1.47 vwxyzA	1.40 xyzA	0.77 DEF	0
AA035	2.26 hijklm	2.93 a	2.93 a	2.93 a	1.40 xyzA	0
AA036	0.53 FGHIJKL	1.83 pqrst	2.23 ijklm	1.20 AB	0.87 CDE	0
C3	2.93 a	2.93 a	2.93 a	2.93 a	1.03 BCD	0.53 FGHIJKL
WGNT6	2.93 a	2.93 a	2.93 a	2.93 a	0.77 DEF	0.33 JKLMNO
WGNT9	2.93 a	2.93 a	2.93 a	2.93 a	1.41 xyzA	0.57 FGHIJK
PNGU36(1)	2.93 a	2.93 a	2.93 a	2.93 a	1.47 vwxyzA	0.57 FGHIJK
PNGU36(2)	2.93 a	2.93 a	2.93 a	2.93 a	1.37 yzA	0.57 FGHIJK

Growth rate values designated with the same lower and/or upper case letter(s) are not significantly different (*p* < 0.05) according to Tukey-test.

## Data Availability

All the data for this research are available on request from the corresponding author. The ITS sequence data can be found at NCBI website (https://www.ncbi.nlm.nih.gov/) (accessed on 24 December 2022).
